# Accelerating Nigeria towards malaria elimination requires moving away from business as usual: insights from a political economy analysis

**DOI:** 10.1093/inthealth/ihaf113

**Published:** 2025-10-07

**Authors:** Elisabeth G Chestnutt, Stefanie Meredith, Babatunde Ipaye, Dawit Getachew, James K Tibenderana, Ebere Anyachukwu, Timothy Obot, Kolawole Maxwell

**Affiliations:** Malaria Consortium, 244–254 Cambridge Heath Road, London, E2 9DA, UK; Global Health Consulting, Divonne les Bains, France; Peltom Global Services Ltd, Abuja, Nigeria; Malaria Consortium Nigeria, 33 Pope John Paul II Street, Maitama, Abuja, 904101, Federal Capital Territory, Nigeria; Malaria Consortium, 244–254 Cambridge Heath Road, London, E2 9DA, UK; UK Foreign, Commonwealth and Development Office, British High Commission, Plot 1137 Diplomatic Drive, Central Business District, Abuja, Federal Capital Territory, Nigeria; National Malaria Elimination Programme, 23 Adesols Olusade Crescrent, Gwarinpa, Kadobunkro, 900108, Abuja, Federal Capital Territory, Nigeria; Malaria Consortium Nigeria, 33 Pope John Paul II Street, Maitama, Abuja, 904101, Federal Capital Territory, Nigeria; London School of Hygiene & Tropical Medicine, Keppel Street, London, WC1E 7HT, UK

**Keywords:** health policy, malaria, malaria elimination, Nigeria, political economy analysis

## Abstract

**Background:**

Despite global efforts to eliminate malaria, progress in Nigeria has been slow. Political economy analysis (PEA) is increasingly being used to identify how political economy influences effective program implementation. Here we apply PEA to the malaria program in Nigeria to understand the contextual factors that have hindered progress.

**Methods:**

A desk review and stakeholder mapping were carried out to identify the relevant actors in the malaria sector. Semi-structured, open-ended interviews were conducted with key influencers and high-level managers. Data were analysed and grouped thematically into factors affecting resource allocation and factors affecting the use of allocated resources.

**Results:**

Factors affecting resource allocation included malaria receiving limited attention and resources due to low prioritisation by federal, state and local governments; weak advocacy from citizens, which means malaria elimination is not an electable issue for politicians; and no direct communication channels between the malaria program and key decision-makers. Factors affecting the use of allocated resources included poor coordination between multiple partners working on malaria.

**Conclusions:**

Achieving meaningful progress in malaria elimination in Nigeria requires predictable financing from sustained political will. Demand from citizens is essential to encourage political prioritisation. Programs and partners must also be better coordinated to maximise impact with limited resources. Establishing high-level malaria advocacy groups and integrating malaria priorities into the national development plan would support these efforts.

## Introduction

Since 2000, there has been a concerted global effort towards eliminating malaria. For much of the last 2 decades there has been substantial funding from international donors, allowing the implementation of technically sound malaria programs in many countries. This funding has resulted in 12 countries being certified malaria free and a further 13 achieving zero indigenous cases for 3 consecutive years.^1^ However, despite these success stories, malaria continues to be a leading public health issue in sub-Saharan Africa, with the region accounting for approximately 95% of malaria cases and 96% of deaths from malaria globally.^1^

In Nigeria, although the National Malaria Strategic Plan (NMSP) 2014–2020 set the goal of eliminating malaria by 2020, progress has been slow, and it remains the country with the highest rates of morbidity and mortality from malaria.^2^ One reason is that the ambitions of the National Malaria Elimination Programme (NMEP) are not being matched by government spending. In 2017, although approximately 1.7 trillion naira (US$3.8 billion) was spent on malaria—equivalent to 40% of all health expenditure—only 8% of this was from the Nigerian government.^3^ In fact, the majority (88%) of malaria spending is from households, through out-of-pocket expenditures, which places the financial burden of malaria on citizens.^3^ Providing adequate resources to support the elimination goal of the NMEP is critical for creating a sustainable malaria program in Nigeria.

In 2018 the World Health Organization (WHO) launched the ‘high burden to high impact’ approach, in the 11 highest-burden countries, to reignite progress to eliminate malaria. This approach recognised that malaria will not be eliminated through technological solutions alone and identified political will as necessary for success.^4^ Evidence has shown that well-designed and executed political economy analysis (PEA) has enabled policymakers and implementers to move beyond technical issues in project and program management to identify the interests, incentives and institutions that can enable the change on which effective development depends.^5^ PEA typically involves document review, mapping of key stakeholders and collection of primary data through focus group discussions and interviews with relevant stakeholders. The findings of a PEA are then used to inform programming to achieve greater outcomes.^5^

Donor agencies and development organisations are increasingly using PEA to identify the contextual factors—political, economic, social and cultural—that drive change, influence decision-making and shape power relations. As part of the UK Foreign Commonwealth and Development Office (formerly the Department for International Development [DFID])-funded Support to the National Malaria Programme 2 (SuNMaP2), we designed the PEA outlined below to understand how the external environment influences decision-making and impacts the success of the malaria program in Nigeria. The PEA study was carried out in the Federal Capital Territory of Nigeria and six SuNMaP2 program-supported states. This article focuses on the findings from the PEA conducted at the federal level.

The PEA aimed to understand the factors affecting the implementation of the NMSP 2014–2020, with a focus on national-level decision-making. This study also discusses the opportunities to unlock progress that can help to inform future NMSPs and applications to the Global Fund to Fight AIDS, Tuberculosis and Malaria (Global Fund). Finally, this article discusses how the findings have informed the NMEP’s planning and implementation, and the actions that have been taken to increase advocacy and domestic financing for malaria since the study’s completion.

## Methods

### Approach and conceptual framework

This PEA draws elements from the DFID Drivers of Change approach, Edelman’s sourcebook on sector-level political economy approaches and the Three Layers of Problem-Driven Governance and Political Economy Analysis by Fritz et al.^6–8^ The aim was to identify the areas that need to be strengthened, revised or changed to promote a sustainable national malaria program able to achieve its goals.

**Figure 1.**
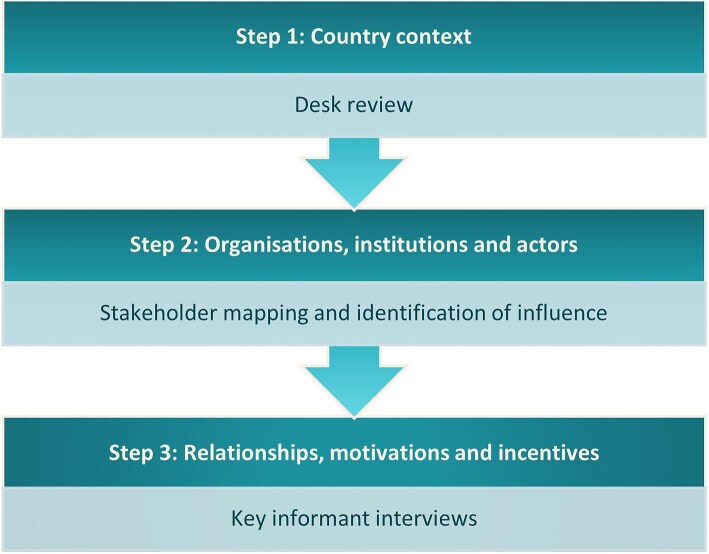
Conceptual framework for the PEA.

A simplified framework (Figure 1) was used to review the contextual factors to establish how policymaking, decisions, implementation and performance in the health sector, particularly for the malaria subsector, are influenced; identify key influencers of change to establish who has power and over what decisions; and assess the perceptions of the actors to understand how the power, interests, incentives, values, beliefs and ideologies of different stakeholders shape the context for the implementation of the malaria elimination program.

### Study setting and time frame

**Figure 2.**
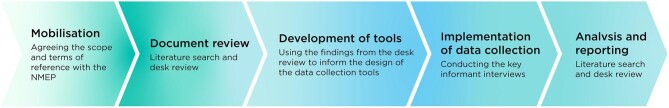
The process followed to conduct the PEA.

This study was carried out from April to November 2020. A mixed methods approach was used, employing qualitative cross-sectional research comprising a desk review, mapping of key stakeholders and institutions and collection of primary data through key informant interviews (Figure 2).

### Study design

#### Desk review

A review of peer-reviewed and grey literature was carried out in May and June 2020 to identify and describe the current political and economic context of Nigeria’s malaria program and inform the research design. To identify articles for inclusion, we searched PubMed (2000–present), Google and Google Scholar (2000–present) and the websites of the World Bank and WHO. In each database we searched for combinations of the following terms: ‘political economy’, ‘analysis’, ‘health’, ‘Nigeria’, ‘Africa’ and ‘malaria’.

#### Selection of key informants

The sample frame for key informant interviews was developed from the desk review and stakeholder mapping. The participants included elected and non-elected political leaders with direct and indirect influence on the health sector, legislators involved in budget making and health sector or malaria oversight, sectoral technical mandate managers, policymakers, agents of relevant departments and institutions, implementing partners, funding partners, civil society organisations and private-sector stakeholders. Participants were recruited between July and August 2020.

#### Data collection

Semi-structured and open-ended interview guides (Supplementary Text S1) were developed to address the research objectives. In-depth interviews of key informants were carried out by three senior consultants from August to October 2020. Interviews were due to be carried out face-to-face, however, due to the coronavirus disease 2019 (COVID-19) pandemic, many of the interviews were conducted virtually using Zoom, Skype and telephone. Participants gave written informed consent and confidentiality and anonymity were offered.

### Data analysis

A general inductive and deductive approach was employed for the analysis of the qualitative data collected during the key informant interviews. This involved manually condensing the raw textual data into a summary format, establishing the main thematic issues and links between the information and study objectives and then identifying from the data which factors interviewees deemed to have a greater impact on resource allocation and programmatic sustainability. As far as possible, data from the various sources was triangulated to increase reliability and validity.

## Results

### Desk review

The literature reviewed included national documents relating to national health policies and strategies, national guidelines, SuNMaP2 program documents, reports from international organisations, media articles and peer-reviewed publications on the political economy of health broadly, in Africa and specifically in Nigeria. No peer-reviewed literature was identified where the primary goal was to explore the political economy of the malaria program in Nigeria. However, one article discussed political will, leadership and financing as one of the challenges to achieving malaria elimination in Nigeria and another explored decision-making around vector-control policies in Nigeria.^9,10^

### Stakeholder mapping

Key influencers were identified through the desk review and the research team’s existing knowledge of the health sector in Nigeria (Table 1). The influence of each actor on health-sector decision-making was determined by information found in the desk review and triangulated with information given by participants during the interviews. Key information for determining influence included who has the authority to make decisions over what resources and whether the influence is direct or indirect.

**Table 1.** The results of the stakeholder mapping demonstrating the key influencers, level of influence and a description of their influence.

Key influencer	Influence on health
Governance, policy, leadership and domestic health financing	
President	Can allocate more resources through the health-sector budget and through service-wide votes at his discretion or according to the political party’s agenda
National assembly: Senate Committee on Appropriation Senate Committee for Health and Primary Health Care	Can review and amend allocations based on federal Ministry of Health submission Relies on feedback during public hearings The Senate Committee for Health is guided by information from constituents and relevant stakeholders
House of Representatives: House Committee on AIDS, Tuberculosis and Malaria, Health and Primary Health Care	Can influence allocation based on targeted advocacy Determines what is referred to appropriation committee Can be influenced by constituents and sectoral stakeholders
Federal Ministry of Health	Responsible for prioritisation—based on evidence with data through the NMEP Responsible for policy formulation, planning and budgeting at federal level
NMEP	Responsible for evidence-based policies and budget implementation at federal level Ensures collaboration with broader health systems, civil societies and community system
Office of Budget and National Planning	Informs ministries, departments and agencies on priorities for allocation Provides ministerial budget envelope
Ministry of Finance, Budget and Planning	Coordinates the engagement of international partners and approve bilateral agreements
Department of Planning Research and Statistics	Coordinates local partners Provides strategic information
State governors	Influences priorities, budgets etc
House of assembly committee on health	Influence the allocation of resources to health and malaria
Service delivery	
State Ministry of Health	Responsible for resource mobilisation and allocation, policy adaptation and planning and implementation support to the subnational levels
State Malaria Elimination Programme	Responsible for the implementation of the operational plan Supports state ministry of health’s service delivery at facility and community levels
Other line ministries	
Ministry of Environment, Ministry of Women’s Affairs, Ministry of Education	Jointly formulates collaborative programming frameworks Supports strategy and plan implementation
Other key agencies	
National Primary Health Care Development Agency, Basic Health Care Provision Fund, Saving One Million Lives	Jointly formulates collaborative programming frameworks Supports implementation at primary healthcare level and integration of activities for efficiency and effectiveness Supports implementation
World Health Organization	Establishes integrated global guidelines, develops country-level planning tools and monitoring and evaluation frameworks Supports country’s efforts towards achieving global technical strategy targets for malaria
Donors	
UK Department for International Development^a^ Bill & Melinda Gates Foundation World Bank USAID President’s Malaria Initiative for states Global Fund	Provision of funds for prioritised gaps Resource allocation for specific programs and sometimes in specific locations Donor funding often predetermined and sets priorities Some donors as influence agenda setting in the states where they work (the President’s Malaria Initiative and Global Fund support 11 and 13 states, respectively)
Non-state actors	
Non-governmental organizations, implementing partners	Support policy, strategy, planning and implementation and service delivery
Traditional and religious leaders	Facilitate community awareness and acceptance. Champion malaria response at the community level
Civil society/community	Advocate for resources and promote access to quality healthcare and services Supports communities to hold authorities accountable Builds awareness of health as their right
Media	
Journalists/media outlets	Disseminate information Hold leaders accountable

a Since completion of the study the UK Department of International Development has been merged with the UK Foreign and Commonwealth Office. This is now collectively known as the Foreign, Commonwealth and Development Office.

### Key informant interviews

Interviews were conducted with 21 stakeholders (Table 2). Respondents were from the health sector (leaders and managers), legislature, partner organisations (implementing and funding) and the private sector. Interviews were not conducted with stakeholders from the National Malaria Elimination Programme or other line ministries due to availability.

**Table 2.** Number of respondents interviewed by sector.

Category	Participants, n (%)
Governance, policy, leadership and domestic health financing	6 (29)
Service delivery	2 (10)
Other line ministries	0 (0)
Other key agencies	1 (5)
Donors	10 (48)
Non-state actors	1 (5)
Media	1 (5)
Total	21 (100)

### Key findings

The findings from the interviews are grouped under two key areas: factors affecting the allocation of more resources and factors affecting the use of existing resources. These areas are subdivided into the underlying causes.

#### Inadequate resources due to lack of prioritisation

A key theme that emerged was that the impact of the malaria program is constrained by limited allocation of resources, due to the low prioritisation of malaria by the government. Respondents were unanimous in voicing that the country needs to put resources on the table for malaria, and several respondents reported that currently there is not enough political commitment, especially at high levels within the government, for malaria elimination in Nigeria.

The ministry has not put emphasis for malaria as a priority, what they put in the budget doesn’t count as a priority.

Respondents also reported that the allocated budget release is poor, often counterpart funding for Global Fund programs is not released and there is limited commitment at the state level for co-financing. This demonstrates the lack of funding is a long-standing issue at the federal and state levels.

We [Nigeria] have always struggled as a country to meet the 15% counterpart GF [Global Fund] contribution.

When delving into the reasons for the lack of prioritisation and subsequent financial commitment, participants posed several reasons.

##### Lack of health economic link.

First, several key informants emphasised that there is a lack of knowledge within the government around the relationship between health and economic outcomes. Participants said a way to incentivise the prioritisation of malaria elimination would be to demonstrate the link between healthy communities and economic stability and growth.

Even at the political party level it would be good to educate them about the relationship between health and the economy.

##### Not politically attractive.

In addition, respondents said the lack of prioritisation of malaria, and health programs in general, is because health is not seen as a priority politically or by the citizens. Respondents said health is seen as a humanitarian issue and there is no real demand or expectations for health services by the population.

Health has low political priority—no demand or expectation by the population.

Several participants reported that malaria is seen as a normal situation and citizens are not aware that they can, and should, advocate for the government to provide better health services.

They [citizens] should demand for accountability and represent the voice for quality service, service coverage and promote use of services.

The limited demand from citizens means that malaria is not seen as an issue that will win votes and therefore does not feature in political campaign manifestos, nor is it ranked highly on the political agenda. In comparison, the bulk of the federal health budget goes to tertiary care as well as to the National Primary Health Care Development Agency (NPHCDA) for immunization and other services at the primary healthcare level because these are popular with the political class.

##### Lack of demand from citizens, civil society and the media.

Respondents said the lack of demand from citizens is fostered by a weak civil society voice for malaria.

Civil societies in Nigeria are no longer active like in the military era, they are not holding us and the partners to account anymore. This for me is important for our system to grow and to keep us on our toes.

Participants reported that unlike human immunodeficiency virus (HIV)/acquired immunodeficiency syndrome (AIDS), malaria does not have a strong civil society group advocating for the issue. One respondent said although networks such as the Civil Society for Malaria Control, Immunization and Nutrition (ACOMIN) exist, the advocacy carried out by these organisations is directed at the Global Fund rather than the federal and state governments for domestic resource mobilisation. In addition, it was reported that ACOMIN does not carry out awareness-raising or demand-creation activities in the population. One respondent said that ACOMIN has little influence with the government and the private sector, which may explain why they do not focus their efforts on domestic-resource mobilisation.

Without doubt, the civil society needs to be repositioned for accountability and community advocacy.

Several respondents gave suggestions of how civil society could be strengthened. One respondent said they should build on the momentum of other successful actors and programs.

Nigeria also needs to create a sense of urgency for malaria elimination leveraging on the momentum created by the polio elimination.

Another participant mentioned the Health Reform Foundation of Nigeria as a good example of an influential civil society organisation that has pushed for some changes in the health sector; however, at present, malaria is not one of the issues that the organisation supports.

Some respondents said engaging the media was a good way to raise awareness and create demand, but recognised that the media is dominated by politics and airtime requires funding as journalists will only take paid assignments.

The media in Nigeria is dominated by politics and now by COVID-19.

One participant highlighted that there is an opportunity for citizens and civil society to advocate for malaria during the budget preparation public hearings. These sessions are also carried out for all sectors and provide a window to engage with relevant committees and make the case for malaria.

##### A lack of advocacy from the NMEP.

**Figure 3.**
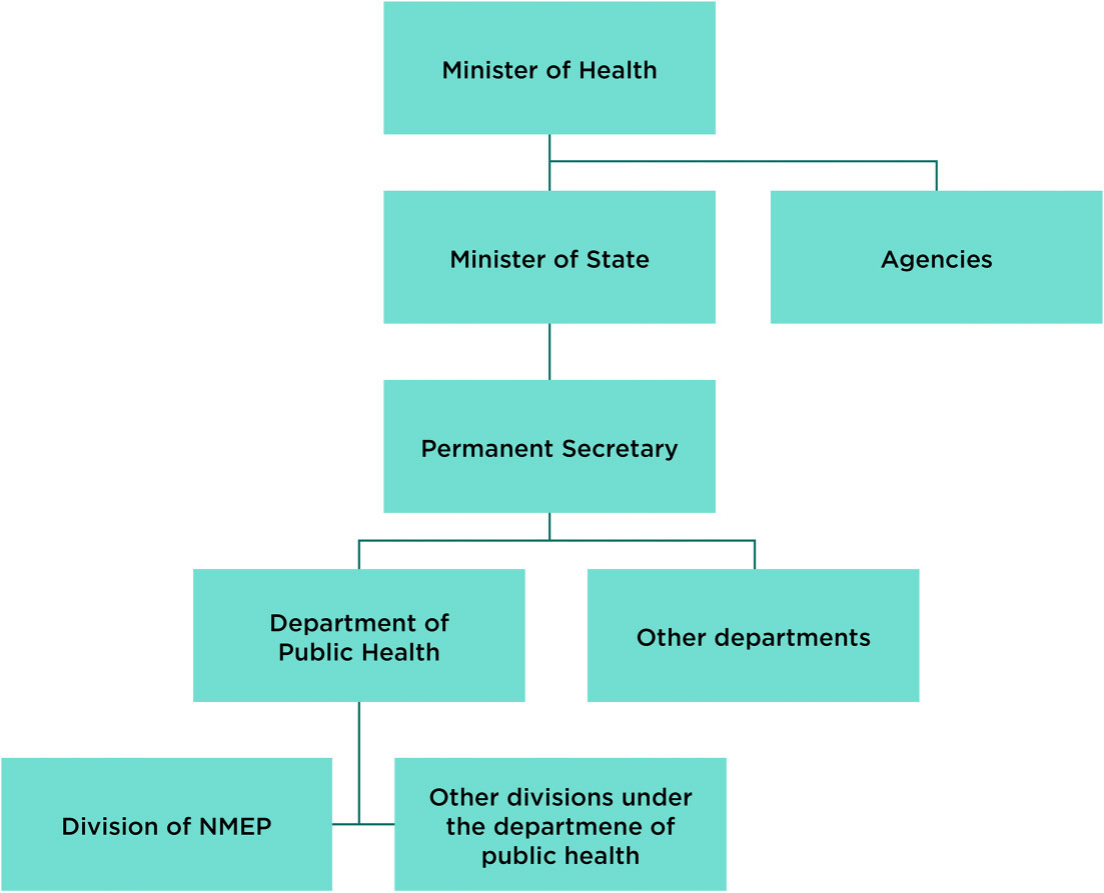
An organogram of the Nigerian Ministry of Health.

Respondents reported that priority setting and budget allocation for health is the responsibility of the Federal Ministry of Health, who make decisions based on the level of advocacy, communication and evidence available. Respondents unanimously agreed that the malaria program, and its partners, do not carry out well-targeted consistent advocacy to politicians and the national assembly. Some participants said this was a structural issue and although the NMEP leadership is technically strong and competent, it is not structurally well positioned to advocate for malaria because there are four levels of reporting between the NMEP and the Minister of Health (Figure 3). Respondents said this makes it difficult for the NMEP to advocate for an increased budget from the Minister of Health, as there is little direct interaction between the two departments.

The NMEP appears detached from the FMOH—it looks like an agency when it isn’t one.

Respondents said that because the malaria program receives external funding from donors, many view it as having agency status. However, a key difference is that the NMEP do not have full control over where this money is allocated. In addition, unlike the NMEP, agencies like the NPHCDA have an executive head who can directly advocate to the presidency and parliament for funding.

Those with agency status have more control over their mandates.

Other respondents reported that advocacy is carried out by the NMEP, but it is limited by poor-quality evidence. It was reported that there is an overreliance on survey data, which only provides a snapshot in time, and not the current situation. Furthermore, participants said the WHO’s approach to use modelling often leads to wrong estimations and routine case data does not accurately reflect the situation, as data are only reported from public health facilities and around half of the population use health services outside the public sector.

There is an over reliance on survey data which does not give you the insight into what is happening on the ground right now.

##### Availability of external funding.

Participants also mentioned that the historical reliance of the malaria program on donor funding disincentivises government spending in this area. Even if malaria was viewed as a priority, the government has limited resources, so if there is funding from another source there is no need to allocate domestic funding. The historical funding has also degraded government ownership of the program, with some respondents commenting that partners are the ones who make decisions on what gets funded. Another participant commented that even as funding from donors has decreased, it has been difficult to change this narrative.

Partners tend to drive things through.

In addition, respondents said the overreliance on donor funding is a key factor that limits the sustainability of the malaria program, as funding can come to an end or be reduced or removed with little notice.

#### Inadequate resources due to dilution of budget requests

A further issue raised by participants is that the administrative distance between the NMEP and the Minister of Health (Figure 3) allows the budget to be watered down as it passes through the other departments. Therefore, the budget requested by the NMEP or State Malaria Elimination Programme (SMEP) may be altered by the Department of Public Health or Permanent Secretary based on their own views of what is needed.

Budget allocation really depends on who is sitting at the helm of affairs at state level.

Respondents also reported that when the Minister of Health reviews the budget they may have little context and remove budget lines they believe are not necessary without discussion with the NMEP.

#### Inefficient use of allocated resources

Participants also said that progress on malaria could be improved if existing resources were used more effectively. A lack of coordination and collaboration was given as a key reason for the inefficient use of the existing allocation for malaria. Several key non-state actors discussed how a health systems’ approach is needed to address health financing as a whole rather than the financing of individual diseases and programs.

Relative to other sectors, the health sector is relatively well allocated—what we need to focus on is the efficiency in the use of resources available to the health sector.

##### Lack of coordination between the NMEP, other ministries and state-level activities

First, respondents reported that the Ministry of Health is the only ministry that works with the NMEP, and the NMEP Coordinator is not empowered to collaborate with other ministries, e.g. the Ministry of Education and Ministry of Environment. Participants also mentioned that there is a lack of coordination between the NMEP and SMEPs and the division of responsibility between federal- and state-level programs is not well defined.

There is little or no cross-sectorial collaboration with other relevant ministries. Specific example of how the ministry of environment is not present in any of the health programmes—but this is across the board—NMEP functions only within the MOH [Ministry of Health].

Several participants also said there is a need for malaria to be more integrated into primary healthcare and this could provide an opportunity for cost-sharing in areas of overlap, e.g. training community health workers to treat a variety of health conditions instead of solely malaria.

The current disjointed investments across states is not working, there is a need to bring implementation of programmes to the PHC [primary health care] platform.

One participant stated that there is an opportunity for the NMEP to collaborate with the Governor’s Forum, which implements the Basic Health Care Provision Fund, to explore how malaria can be streamlined into it.

##### Lack of coordination between partners and the health sector

In addition, participants highlighted the lack of coordination between partners working on malaria with each other and the health sector. Participants mentioned that programs for HIV/AIDS, malaria and tuberculosis are all still vertical—in management, structure and funding—and there is little or no cross collaboration, which is a major barrier to the development of sustainable national and state malaria elimination programs. Participants said the government should do more to coordinate partners working in the malaria sector to ensure they are aligned.

National government is not strong enough in its expectations and messages to donors. It should be insisting on better coordination.

##### Exclusion of the private sector

Finally, participants also mentioned a lack of coordination with the private sector. This was seen as an unexplored opportunity, as there are a number of private-sector foundations supporting malaria who are not included in the NMEP planning sessions. This isolates the private sector and leads to decisions that could damage the commercial market, which results in a situation where the private sector is unwilling to participate. In states such as Lagos, Jigawa and Sokoto, a drugs revolving fund system scheme has been shown to work effectively. However, a strategy is needed to ensure these schemes are competitive with retailers, which requires manufacturers and states to be linked together to drive down cost of malaria commodities. Likewise, to improve distribution of malaria commodities from the central medical stores it was suggested that the government should partner with the private sector to set up efficient push systems for ordering, tracking and delivery of commodities.

## Discussion

This PEA found that progress to eliminate malaria has been slow in Nigeria due to ineffective mobilisation and use of resources by the malaria program. The inference from the findings is this has resulted in major gaps in the provision of health services, with inequity in human and material resources across states and preventing access to malaria and other health services for many of the population. Several underlying factors have inhibited effective mobilisation of resources, including the structural placement of the NMEP, the historical reliance on external funding resources and the acceptance of malaria as a fact of life by politicians and the public. In addition, coordination of the existing resources by the NMEP has been weak, which has resulted in inequitable distribution of resources and duplication of effort.

Our study confirmed that political will is a precondition for resource allocation and political interest is driven by advocacy to the political class. This is well documented in other studies and included as a key pillar of the WHO’s high burden to high impact approach.^4,9–11^ Despite this point being well documented, few studies have described in detail how to increase political will. Our work demonstrates that PEA is a useful tool to aid understanding of the factors that drive political will. We found that since >70% of malaria deaths occur in children <5 y of age, who are unable to advocate for themselves, the disease has historically not been viewed as an electable issue.^1^ In comparison, advocacy is much higher for tuberculosis and HIV/AIDS, as they result in lifelong conditions affecting adults who are able to advocate and vote. A study in Uganda found that when rural communities were able and willing to use their voice, they were successful in influencing the issues politicians were interested in.^12^ This highlights the need for more support for rural communities to build a strong voice advocating for malaria elimination.

In addition, the work of the NMEP is negatively influenced by its placement in the institutional structure of civil service, which makes it difficult to advocate for higher prioritisation and increased resources. The NMEP is situated four reporting levels away from the Minister of Health, where budget decisions are made (Figure 3). This results in a lack of direct dialogue between these levels of authority in the government and a lack of transparency and negotiations around budgeting decisions. In contrast, if the NMEP had an agency status, akin to that of the National Agency for the Control of AIDS, it would be easier to advocate for resources as they would report directly to the Minister of Health or a higher authority.

Furthermore, the benefits of eliminating malaria have not been effectively disseminated to politicians, and with funding available from external sources, there is little incentive to provide more resources to the NMEP. Implementing partners should also work with the NMEP and civil society to effectively communicate coordinated messages that are backed by evidence, including providing legislators with data that demonstrate the link between the burden of malaria and economic development. Strengthening surveillance systems to collect high-quality data is essential for building evidence for both advocacy and contextualised data-informed decision-making for targeting interventions.

**Figure 4.**
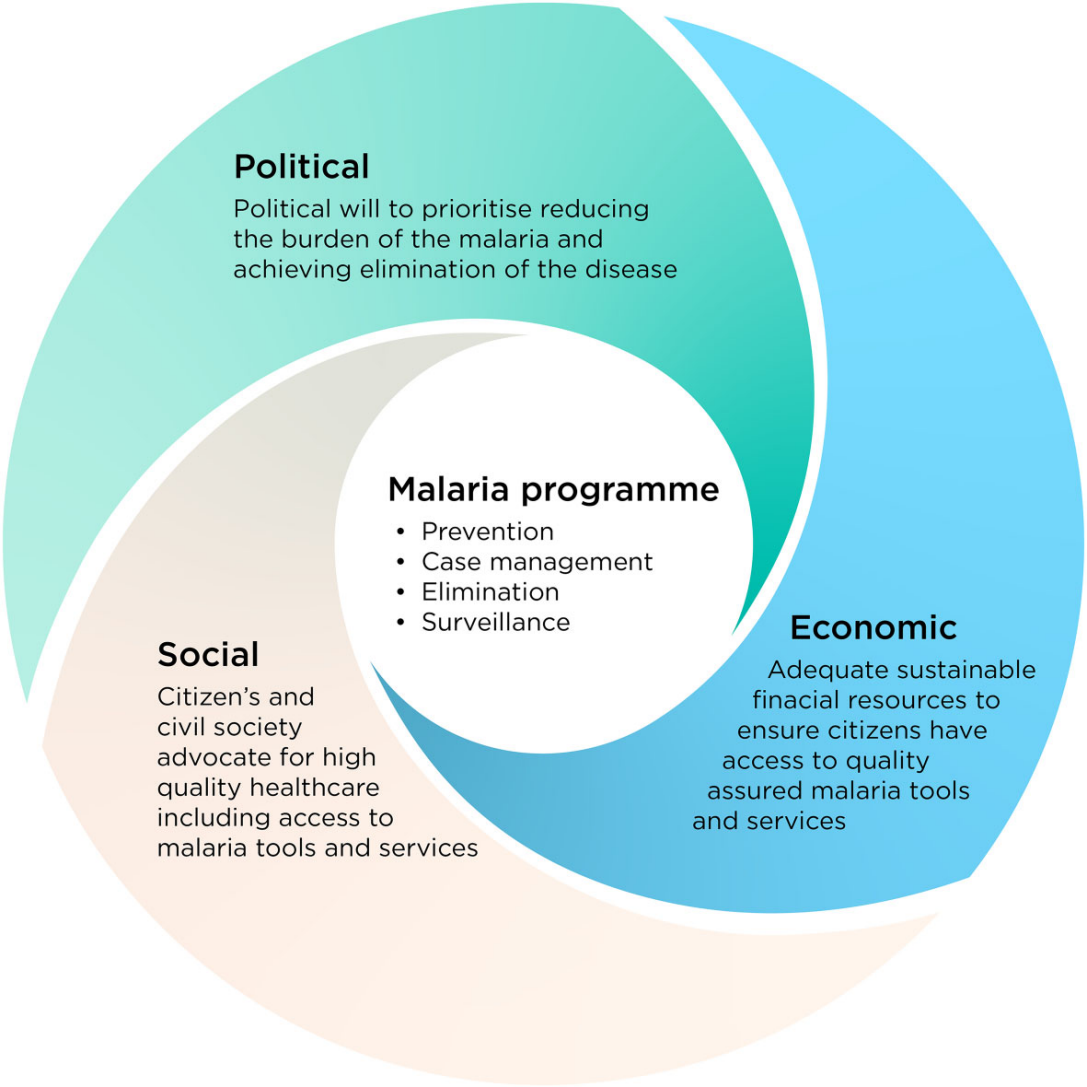
The necessary political, economic and social conditions to support a sustainable NMEP.

Creating a sustainable malaria program requires strong advocates to incentivise politicians to allocate resources, which can then be used to build evidence for elimination (Figure 4). The low prioritisation of malaria, historical donor reliance and resulting lack of funding for the program creates a cyclic effect where funding is not available to improve the quality of data collection and reporting that could be used for advocacy and communication to create demand among the population and politicians.

In addition to the conditions outlined in Figure 4, the malaria program also needs to play a stronger role in coordinating with other ministries, partners and the private sector to effectively channel resources. Historically the NMEP has not coordinated with the Ministry of Environment, which is responsible for leading investments in environmental management and can impact vector control activities. Stronger ties and common goals between these ministries and departments will benefit the work of the NMEP, as working in isolation can negate efforts if not aligned.

Coordination and communication between partner organisations has also been poor, due in part to weak stewardship from the NMEP. Historically, partner organisations have provided the funding and therefore have had influence over where it should be targeted. This has led to situations where multiple partners are working outside of institutional structures and on their own agenda, leading to duplication of effort and leaving gaps elsewhere. This was a similar finding in the countries studied by Parkhurst et al.^13^ and was highlighted as a key action point in the Paris Declaration on Aid Effectiveness.^14^ The NMEP must have a strong coordination role to ensure partners are aligned with the NMSP. In addition, the government should provide meaningful co-funding to support the NMEP to play a greater role in where resources are targeted.

Finally, it was found that the private sector, where almost half of Nigeria’s citizens seek healthcare, is not included in malaria planning meetings.^15^ Failing to include the private sector can lead to further inefficiencies and result in poor-quality services being delivered to citizens. These findings are consistent with those of Parkhurst et al.,^13^ who found that engaging with the private or non-governmental sectors was an important consideration for the malaria programming. The creation of public–private partnerships can help to share knowledge and coordinate activities across sectors to avoid duplication, which accelerates efforts towards elimination.

The findings of the study are important given the current financial climate. The NMEP and the state counterparts have historically relied on funding from international donors, such as the Global Fund, which accounts for 40% of international malaria spending.^1^ However, in recent years donor funding for malaria globally has decreased and the difference between the resources needed and the resources available is growing, increasing from a gap of US$2.6 billion in 2019 to US$3.8 billion in 2021.^1^ The increasing funding gap has correlated with stalling progress towards global malaria targets. Between 2000 and 2015, when funding was more readily available, the global mortality rate from malaria was halved. However, since 2015 it has fallen by <2%.^1^ A similar trend has been observed in Nigeria, where malaria prevalence fell by 36% between 2010 and 2015 and only 19% between 2015 and 2021.^16^ The COVID-19 pandemic has further constrained funding and some donors have reduced spending on overseas development assistance.^15^ Changes in political leadership in the USA has also resulted in the removal of funding and closure of health programs across the globe. New technologies, such as next-generation nets and vaccines, can help to accelerate progress towards malaria elimination, but they are comparatively more expensive than existing products. This could lead to trade-offs in coverage if funding is not increased to meet demand.

### Outcomes

Since the conclusion of this study and the dissemination of the findings, several actions have been taken to address the challenges identified from the PEA. The NMSP 2021–2025 has been developed, which aims to improve funding sustainability for malaria control by ensuring at least 25% of funding annually is from predictable and innovative sources.^17^

To support this ambition, in August 2022, the Nigeria End Malaria Council, a public–private partnership, was established to bring together the government, private sector and civil society. The council provides a platform to advocate for more funding to protect and sustain progress against malaria and to improve coordination between partners.^18^

In 2023, President Bola Ahmed Tinubu launched the Presidential Initiative for Unlocking the Health Value Chain (PVAC) to encourage new investment to transform Nigeria’s healthcare delivery system.^19^ The same year, the Coordinating Minister for Health and Social Welfare established the first sector-wide approach (SWAp) in the health sector in Nigeria.^20^ The SWAp aims to align all stakeholders under one strategy plan and budget to increase transparency and accountability in the health sector.

In addition, in May 2024 the NMEP, together with partners, co-organised a high-level roundtable, Rethinking Malaria Elimination in Nigeria, which led to the establishment of the Advisory on Malaria Elimination Nigeria (AMEN) and the Ministerial Task Force on Malaria in Nigeria. The AMEN group meets twice a year to review programmatic evidence and approve guidance on best practices for malaria elimination. The Ministerial Task Force works closely with the Ministry of Health and development partners to ensure AMEN’s recommendations are effectively implemented. In addition, at the inaugural meeting of AMEN the Minister of Health and Social Welfare, Muhammad Pate, highlighted the link between malaria and economic development in the country, citing the disease is responsible for US$1.1 billion in annual losses to Nigeria’s gross domestic product.^21^

Most recently, the federal government has launched a technical working group for AIDS, tuberculosis and malaria to strengthen the coordination between programs and agencies working to reduce these life-threatening diseases.^22^ Furthermore, the 2025 budget allocations from the Ministry of Health have budgeted for the malaria vaccine as a new technology that is separate from the allocation for other malaria commodities. This decision was taken within the context of huge media interest in the malaria vaccine and a backdrop of demand from citizens.

### Limitations

The aim of this study was to assess the external factors that influence the work of the NMEP and did not explore in detail the factors within the NMEP that help or hinder progress. Future PEA could explore the factors within the NMEP, e.g. the organisational and institutional capacity, to determine what impact this has on successful implementation of the NMSP.

This study was carried out during the COVID-19 pandemic. This impacted travel, meeting restrictions and infection control precautions, which limited the scope and depth of the PEA. The planned consultative meetings with key stakeholders in the PEA and in-person key informant interviews had to be adapted to the context and most of the key informant interviews were conducted virtually. Key informant interviews are confidential in nature, so usually necessitate face-to-face meetings. The information obtained through telephone, Zoom or Skype calls gave less depth, particularly for sensitive issues, and triangulation of information was challenging. In addition, the members of the NMEP were invited to take part in the key informant interviews; however, no one was available to participate. The NMEP is a key actor with the PEA and therefore their absence leaves gaps in the findings.

A limitation of conducting PEA is the findings only represent a snapshot in time and the opinions of those included in the interviews. The institutional arrangements, influences, actors and motivations that make up the political economy change over time. For this reason, ideally, PEAs should be conducted frequently to keep up with the fluidity of the political economy itself and those using the findings of a PEA should consider how those included and absent from the interviews may affect the results.

## Conclusions and recommendations

Since the findings of this PEA, new initiatives have been established that will support Nigeria’s transition to country-led sustainable financing of the malaria program. However, there will continue to be factors that influence and impede progress as the political economy changes. This study demonstrates that a PEA, grounded on a scientific approach and robust methodology, and contextualized for malaria programming, can serve as a key tool to inform efforts to improve the effectiveness and sustainability of a national malaria program.

Several recommendations can be made from our findings. First, PEAs should be conducted frequently to stay aligned with changes in the political economy. We suggest this should take place at a minimum at the end of each strategic plan to inform the next, however, it may be useful to also conduct a PEA at the midpoint of each strategic plan implementation. For this reason, capacity must be built into the national and, where possible, the state malaria programs so they are able to conduct these assessments. Second, if international funders and partners are aware of the political economy when supporting programs, they can ensure they work in harmony with it and their actions promote sustainability Third, if international partners understand the political economy they will be better informed in their efforts with national and other development partners in addressing the critical issues that hinder progress towards malaria elimination. Finally, if the NMEP recognises the institutional arrangements that impact malaria elimination, they can consider how best to reform the system to better engage malaria within the wider health sector and increase the voice of citizens. The WHO’s call to action to course correct the stalled progress in reducing the occurrence of malaria in high-burden countries requires a collective ‘business unusual’ approach. PEA provides a useful approach that can identify new actions that could create value and contribute to more sustainable and accelerated progress towards malaria elimination.

## Supplementary Material

ihaf113_Supplemental_File

## Data Availability

The data are available in the article and its supplementary material.
