# Supplementary material for: Accelerating Nigeria towards malaria elimination requires moving away from business as usual: insights from a political economy analysis
Source: Int Health. 2025 Oct 7;18(3):419–30. doi: 10.1093/inthealth/ihaf113 (PMC13154835; doi:10.1093/inthealth/ihaf113)
Supplement: ihaf113_Supplemental_File [file ihaf113_supplemental_file.docx]

## **Interview guide for national stakeholders**

N.B. This is a guide – not all questions will be relevant, and many of the sub –questions can be used as prompts. Remind Interviewees that this is a review, not an evaluation, and the interview may provide useful lessons learnt as well as identifying any weaknesses that need to be corrected in order for the programme to reach its objectives. Ask politely if you can record the interview to ensure accuracy. BOTH interviewer and interviewee will be required to sit at least two metres apart and wear face masks.

*Introduction:*

*My name is…………..and that of my colleague is………. We have been asked to speak with you by the Government of ………… State and their Malaria programme partner – SUNMAP2. I/we want to thank you for taking the time to meet with me/ us today to give us your insights and perspectives for the malaria Political Economy Analysis review.*

*The purpose of this PEA is to identify the key political factors and assumptions with a potential to affect Malaria control project implementation. The PEA will be used to inform and guide the future implementation and or adaptation that may be needed for changing policy priorities, resource re-allocation, or change of key programme stakeholder.*

*The Scope and Focus of review:*

*1) Levels of interests, incentives, institutions and stakeholders that can impact the malaria programming and it’s* *the effectiveness*

*2) Opportunities for addressing challenges identified*

*3) How to build long term sustainability*

*4) Level of integration of Malaria programming into the broader health system*

*All responses will be kept confidential. This means that your interview responses will only be shared with research team members and we will ensure that any information we include in our report does not identify you as the respondent.*

*Are there any questions or clarifications before we begin?*

**Draft interview guide**

| *Name of Informant(s)* |  |
| --- | --- |
| *Institution/Organisation* |  |
| *Position* |  |
| *Date of Interview* |  |
| *Interviewer(s)* |  |

| Select which questions are appropriate for this respondent, others may be omitted |
| --- |
| 1. Policy and Leadership |
| What kind of support (financial, administrative, leadership) is there for malaria programming at this levels of Government? |
| What are the current roles being played by the different levels of Health governance (at Federal, state and local government level) related to malaria programming (planning, finance, service delivery, etc)? Are these roles in line with existing policy? Why do you say so? |
| What is the nature and the level of the partnership between the malaria programme and other sub-sectors of Health as well as other non-health sectors, if any?  Probe: (ask about DPH programmes (HIV & TB), NPSCMP, NHIS, RMNCAH, NTDs, WASH, and the extent to which they are relevant, sustainable, and have shared values) ? |
| Are there champions within or outside Government who generate interest in the implementation of malaria–related activities? If so, who are they? How effective are they? |
| Are national and sub-national policies developed, updated regularly and adapted or communicated at community level? Why do you say so? If yes, is it effective in creating a shared understanding at all levels? |
| Do you think there are barriers to malaria control policy implementation at national, subnational, local government or and or community-level? If yes, please explain. |
| Do you know if there is a public communication strategy that cuts across all levels? If yes, is it validated and reinforced by key stakeholders? |
| 2. Health priorities and resource allocation |
| Does the NMEP/SMEP programme carry out joint activities with any of the following aspects of your health system? (Select which are appropriate for respondent)  *- Internal coordination based on its Coordination Framework*  *- External coordination based on its Coordination Framework*  *- Coordination with the HIV/AIDS, TB or NTD programme*  *- Coordination with the WASH programme*  *- Coordination with other health programmes (e.g. RMNCAH of NPHCDA or SPHCDA)*  *- Coordination with other ministries (Budgeting, Planning, Finance, Environment,*  *Women Affairs)*  *- Coordination with the Legislatures (Federal, State and LGA)*  *- Monitoring, Evaluation and Learning (DPRS- Federal or state)*  *- Disease Surveillance ( NCDC or Epid department)*  *- Drug procurement, stock management and distribution*  *- Are there mechanisms for receiving feedback? How regular is feedback provided?* |
| Resource generation: How and by whom is the health system funded at the various levels of government? |
| Resource allocation: How are national or state resources allocated across sectors? (tailor to relevant KI)  How are resources shared among departments, programmes, services, locations or communities?  Which are the high priority programmes (well-funded) vs low priority programmes (poorly-funded). Where does the Malaria programme fit in (high or low priority)? |
| How are malaria programme priorities (objectives) set and operationalised (including funding )– at national level, at State level? |
| Do these priorities/objectives get translated into operational plans and corresponding budgets at both national and State level? Explain how. Does the allocation match the budget? Please explain. Is there a resource mobilisation strategy to cover funding gaps? |
| In your opinion, what are the factors that promote or hinder local-level planning, budgeting and resource allocation for Malaria? |
| How do you think local-level planning, budgeting and resource allocation for Malaria control and elimination can be improved? |
| What do you think could be done by the malaria programme differently to enable the government to move forward towards Universal Health Coverage? |
| In your opinion, are there programmatic factors at National/ State/ Local level that will impact financial and programmatic sustainably? What do you think can be done to mitigate or enhance it? |
| 3. Quality of services |
| Are there measures used to ensure the safety and quality of malaria and health services? If Yes, how are they enforced? |
| Performance monitoring/management: What approaches are used to ensure that services are delivered as planned in terms of quality and quantity? |
| 4. Health workforce and quality |
| What is the size and structure of the health workforce? What proportion is involved in malaria programme (a fraction)? What is the quality of staff recruitment? Is staff deployment based on areas of need and/or equity consideration? What are other major Human Resource issues and how do you think this can be addressed?  (e.g motivation and Morale of Heath workers, incentives for staff) |
| Role of Community Health Workers: What is the role of CHWs in the malaria programme? How are they funded who pays their salaries or stipends? |
| Quality of management and leadership: How would you rate (strong, average or weak) the leadership of the malaria programme? Why do you say so? (Ask about stewardship, vision, capacity, responsiveness, etc.) |
| Quality and availability of training and development: Do you think that staff are adequately trained for the tasks they are being asked to perform? What additional skill set would be useful to the staff a sub-national level (be cadre specific)? |
| 6. Delivery systems and supply chain: Sourcing of inputs: How medicines and other supplies are purchased and supplied; availability of commodities |
| Can you describe how the malaria commodity supply chain works? Who funds and purchases the commodities? If commodities are currently being procured by donors, do you know if government (federal and State) presently procures or has any plans for taking over procurement and distribution? |
| Are all the appropriate anti -malarial drugs available? If Yes, are there availability gaps and stock outs? Please explain why you think this is so. |
| Do you think there is sufficient infrastructure and system in place for timely delivery of the anti- malarial commodities? Do you think there is equitable access to health services, particularly for malaria? |
| Availability and use of ITNs/ LLINs – How are ITNs/ LLINs distributed? How can distribution or possession be improved? Are there barriers to more widespread use? Please explain. |
| Do you think there is an appropriate, efficient process for tracking the malaria commodity supply chain and performance? If No, what could be done to improve this? |
| 7. IEC & BCC and media communication - are people well informed? |
| Are malaria control IEC materials and BCC messaging customized to local context languages? Please describe. |
| Is there consistent messaging across the state? Please explain. |
| Is there community acceptance for the malaria interventions? Please explain. |
| How is malaria reported in the media? What impact has it had? Please explain. |
| 8. Private sector involvement |
| Who are the main private sector stakeholders? (Please name them - e.g Extraction industry, finance/banking, media, telecom, food/beverage industry, agro-industry, airlines, hotels, pharmaceutical etc). What role do they currently play? |
| What is their interest in malaria (e.g how does it affect their business, are they interested in partnership benefits and what are these)? |
| How would the private sector become motivated in malaria programming? |
| 9. Civil society |
| Who are the main groups and organisations that represent citizens’ views on, and experience of, the health system and malaria programme and how influential are they? |
| What other organisations, groups or individuals have the potential to support or oppose the malaria elimination efforts? |
| 10. Evaluation and adaptation (evidence base) |
| In your opinion, is information from M&E being used in strategic planning and policy decisions at this level? If so, please explain how – if not, how do you think this could be improved? |
| Do you know if there has been participation by any of the key stakeholders in developing the plan(s) and indicators, targets and milestones? Please explain. |
| Is there a clear designation of ownership and frequency of data collection and reporting? Please explain. |

**Are there any other issues that you would like to raise related to the malaria programme?**

**Success, weakness and threats**

**-**  Can you give 2 things that have worked well in the NMEP/ SMEP programme – and why?

- Can you give 2 things that have not worked well and why do you think that?

- What do think are the main strengths of the NMEP/ SMEP approach?

- What do you think are the main weaknesses?

- In your opinion, what are the main threats that the programme faces and why?

- In your opinion, what are the main opportunities that the programme has not maximized and why?

**Please give 1 recommendation which would improve one or more of the main programme activities given below:**

•Strengthening national and state government stewardship that would increase the proportion of total annual malaria expenditure that comes from domestic sources

•Increasing the availability of antimalarial commodities, so that 70 percent of the population has access to and sleeps under an insecticide treated net

• Contributing to more efficient and equitable malaria prevention and treatment service delivery

• Ensuring that citizens and institutions are informed of malaria prevention and treatment services and all other programme interventions

•Embedding an evidence-based learning environment in the National Malaria Elimination Programme and State Malaria Elimination Programme.

**Thank you for your participation**
